# Formulation, Development, and In Vitro Evaluation of a CD22 Targeted Liposomal System Containing a Non-Cardiotoxic Anthracycline for B Cell Malignancies

**DOI:** 10.3390/pharmaceutics10020050

**Published:** 2018-04-15

**Authors:** Nivesh K. Mittal, Bivash Mandal, Pavan Balabathula, Saini Setua, Dileep R. Janagam, Leonard Lothstein, Laura A. Thoma, George C. Wood

**Affiliations:** 1 Plough Center for Sterile Drug Delivery Solutions, University of Tennessee Health Science Center, Memphis, TN 38163, USA; bmandal@uthsc.edu (B.M.); bpavan18@gmail.com (P.B.); dileep.janagam@gmail.com (D.R.J.); 2 Department of Pharmaceutical Sciences, College of Pharmacy, University of Tennessee Health Science Center, Memphis, TN 38163, USA; ssetua@uthsc.edu (S.S.); lthoma@uthsc.edu (L.A.T.); gwood@uthsc.edu (G.C.W.); 3 Department of Pathology, College of Medicine, University of Tennessee Health Science Center, Memphis, TN 38163, USA; llothstein@uthsc.edu

**Keywords:** AD 198, B-cell malignancy, liposome, nanoparticle, CD22 targeting

## Abstract

Doxorubicin cardiotoxicity has led to the development of superior chemotherapeutic agents such as AD 198. However, depletion of healthy neutrophils and thrombocytes from AD 198 therapy must be limited. This can be done by the development of a targeted drug delivery system that delivers AD 198 to the malignant cells. The current research highlights the development and in vitro analysis of targeted liposomes containing AD 198. The best lipids were identified and optimized for physicochemical effects on the liposomal system. Physiochemical characteristics such as size, ζ-potential, and dissolution were also studied. Active targeting to CD22 positive cells was achieved by conjugating anti-CD22 Fab’ to the liposomal surface. Size and ζ-potential of the liposomes was between 115 and 145 nm, and −8 to−15 mV. 30% drug was released over 72 h. Higher cytotoxicity was observed in CD22+ve Daudi cells compared to CD22−ve Jurkat cells. The route of uptake was a clathrin- and caveolin-independent pathway. Intracellular localization of the liposomes was in the endolysosomes. Upon drug release, apoptotic pathways were activated partly by the regulation of apoptotic and oncoproteins such as caspase-3 and c-myc. It was observed that the CD22 targeted drug delivery system was more potent and specific compared to other untargeted formulations.

## 1. Introduction

Nanomedicines have seen significant advancements in the past few decades and have been actively pursued as a means of providing alternative drug delivery systems for disease targeted therapies. In 1995, Doxil^®^ established the foundation for the potential of nanomedicines being approved by the United States Food and Drug Administration (FDA) [1]. Since then there has been a rapid increase in research on nanoparticulate drug delivery systems [2]. This has led to the FDA’s approval of Abraxane^®^ [3], DaunoXome^®^ [4] and most recently, Marquibo^®^ [5,6]. Nanoparticles have provided researchers with the tools to overcome some of the drawbacks of conventional drug delivery systems, most common of which are adverse effects due to non-specific actions of the drug or the drug delivery system [7,8]. Specific binding nature of ligands can be exploited to target these types of drug carriers to the target tissues or cells [9,10,11,12,13,14]. Antibodies are one such type of ligand that can be conjugated to nanoparticles to aid in targeted drug delivery. Liposomes are by far one of the most commonly used of the nanoparticulate drug delivery systems, commercially and in clinical trials [15,16,17]. Liposomes are biodegradable in nature and are versatile in the drug that can be carried as well as the ligands that can be conjugated to their surface for targeted drug delivery [18,19].

Cancer therapy is one field in which researchers have been consistently exploring for breakthroughs using nanotechnology as their primary tool for targeted drug delivery [20,21]. Current medications are still not sufficient to treat the numerous variations of cancers [21], primarily because each variation of cancer needs customized therapies for each patient [20]. Nanotechnology is an adaptable science that can be used to create tailor-made drug delivery systems for specific malignancies.

B cell malignancies are a type of hematological malignancy that have almost 80,000 new cases every year and claim the lives of almost a third of these [22,23]. The standard therapy for B cell malignancies is CHOP [24], of which doxorubicin is an integral part. The treatment of hematological malignancies presents considerable differences from solid cancers in that a large population of the cancer cells are circulating. In a previous review [25] we had discussed the potential of nanoparticles, such as targeted liposomes, being utilized for targeted therapies for B cell cancers. Drug delivery scientists have worked towards the development of a targeted nanoparticulate system for the treatment of B cell cancers [11,13,14,23,26,27,28,29,30,31]. Most of these groups have utilized doxorubicin [11,14,23,32,33] or vincristine [5,6,34] as the drug of choice. Although nanoparticulate systems for both these drugs are already approved by the FDA [1,6], significant enhancements are still needed in view of the adverse effects associated with the non-specific action of the drug delivery system [20] as well as the inherent toxicity of the drug [35]. This has supported the use of novel molecules that would exhibit more desirable properties than currently approved drugs.

**Figure 1:**
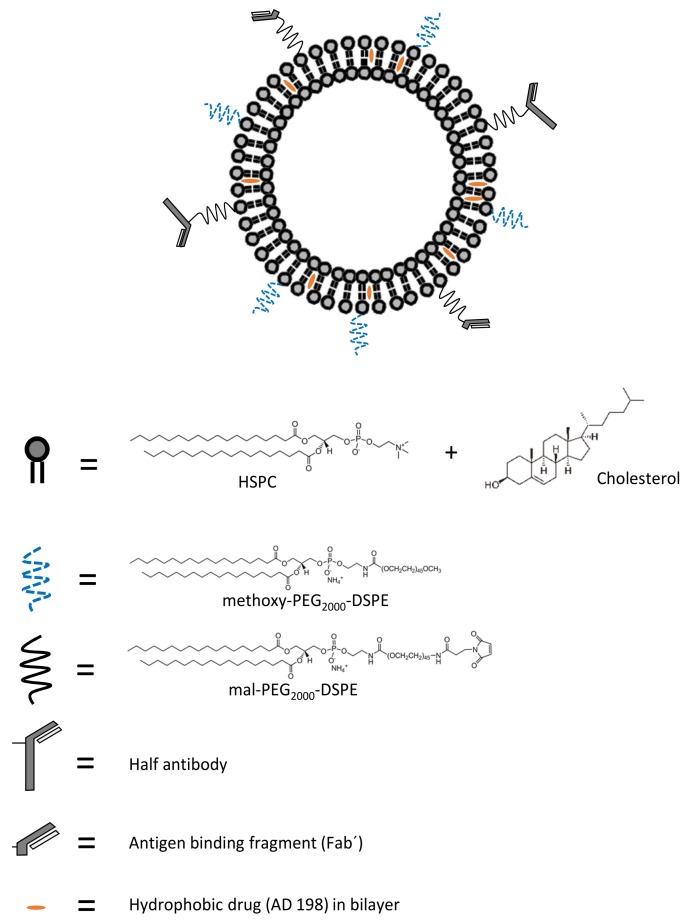
Components of developed long circulating CD22 targeted liposomal AD 198 drug delivery system (HSPC: hydrogenated soy phosphatidylcholine, methoxy-PEG_2000_-DSPE: 1,2-distearoyl-*sn*-glycero-3-phosphoethanolamine-*N*-[methoxy(polyethylene glycol)-2000], mal-PEG_2000_-DSPE: 1,2-distearoyl-*sn*-glycero-3-phosphoethanolamine-*N*-[maleimide(polyethylene glycol)-2000].

Several strategies have been investigated to reduce adverse effects such as the cardiotoxic potential of doxorubicin. One strategy is designing less toxic anthracycline analogues such as epirubicin and idarubicin. However, these analogues only succeeded in delaying cardiotoxic events to higher doses, later stages of therapy or by producing lesser acute cardiotoxicity [36,37,38]. Valrubicin however, is one anthracycline analogue which lacks cardiotoxicity [39]. *N*-Benzyladriamycin-14-valerate (AD 198) is another anthracycline that displays no dose-dependent cardiotoxic properties along with an added cardioprotective action from the damage caused by doxorubicin [40,41,42]. It is a protein kinase C (PKC) activating agent that displays superiority over doxorubicin [41]. It functions by a completely different mechanism of action compared to doxorubicin which has been or is under study by four groups, Cekanova et al., Xie et al., He et al. and Lothstein et al. [40,43,44,45]. AD 198 does not display any significant organ toxicities and is less myelosuppressive compared to doxorubicin [39]. Myelosuppression, even in reduced forms, is debilitating for the patients undergoing prolonged treatment. To cope with the adverse effects such as neutropenia and thrombocytopenia, we have developed long circulating CD22 targeted liposomes loaded with AD 198 (LCCTLA) and have compared their efficacy with long circulating untargeted liposomal AD 198 (LCLA) and free drug [46]. Consequently, the development of a targeted drug delivery system was expected to impose specificity and considerably moderate adverse effects compared to the other two formulations. Figure 1 gives a schematic representation of the developed LCCTLA drug delivery system.

## 2. Materials and Methods

### 2.1. Materials

HSPC (hydrogenated soy phosphatidylcholine), EPC (egg phosphatidylcholine), mal-PEG2000-DSPE (1,2-distearoyl-*sn*-glycero-3-phosphoethanolamine-*N*-[maleimide(polyethylene glycol)-2000]), mPEG2000-DSPE (1,2-distearoyl-sn-glycero-3-phosphoethanolamine-*N*-[methoxy(polyethylene glycol)-2000]) and NBD-PC (12-[*N*-(nitrobenz-2-oxa-1,3-diazol-4-yl) amino] dodecanoyl phosphatidylethanolamine) were purchased from Avanti Polar Lipids, Alabaster, AL, USA, cholesterol, MTT (3-(4, 5-dimethylthiazol-2-yl)-2, 5-diphenyltetrazolium bromide), amiloride, genistein, M-β-CD (methyl-β-cyclodextrin) and chlorpromazine were purchased from Sigma-Aldrich Co. LLC, St. Louis, MI, USA, chloroform, methanol, Whatman^®^ Nucleopore track etched polycarbonate membranes, 200 proof ethanol, 10× PBS (phosphate buffered saline) and HPLC (high pressure liquid chromatography) grade water, Slide-A-Lyzer^®^ MINI Dialysis Devices, 3.5 kD MWCO (molecular weight cut off), 0.5 mL capacity, ammonium hydroxide and 80% formic acid, immobilized pepsin, Thermo Scientific™ CL-XPosure™ Film (X-ray Film), Sepharose CL4B gel filtration gel, anhydrous citric acid, empty PD-10 columns, SDS (sodium dodecyl sulfate), DMF (dimethylformamide), DMSO (dimethyl sulfoxide), 80% acetic acid 1 N HCl (hydrochloric acid) and LysoTracker^®^ Deep Red were purchased from Thermo Fisher Scientific, Waltham, MA, USA, ultrapure nitrogen was purchased from Nexair, Memphis, TN, USA, Sephadex G50 pre-filled macro SpinColumns^®^ and empty macro SpinColumns^®^ were purchased from Harvard Apparatus, Holliston, MA, USA, Total Recovery^®^ HPLC vials were purchased from Waters, Milford, MA, USA, anti-CD22 monoclonal antibody (RFB4) was a generous gift from the lab of Ellen Vitetta, University of Texas, Southwestern Medical Center, Dallas, TX, USA, Amicon^®^ Ultra—0.5 mL centrifugal filters, Ultracel^®^—100 K and Ultracel^®^—30 K were purchased from Millipore, Bellericka, MA, Laemmli buffer and polyacrylamide gels were purchased from Bio-Rad, Hercules, CA, USA, Daudi and Jurkat cells were purchased from ATCC, Manassas, VA, and Vectashield^®^ cell mounting medium with DAPI (4′,6-diamidino-2-phenylindole) was purchased from Vector Labs, Burlingame, CA, USA, Iron oxide nanoparticles were purchased from Ocean NanoTech, San Diego CA, USA, antibodies for c-myc #5605, pAKT #4058, caspase-3 #9662 and anti-mouse secondary #7076 were purchased from Cell Signaling Technologies, Danvers, MA, USA and antibodies for pJNK (sc-571), β-actin (sc-130065) and anti-rabbit secondary (sc-2357) were purchased from Santa Cruz Biotechnology, Dallas, TX, USA. TEM (transmission electron microscopy) sample preparation materials were generously provided by the Imaging Center at the Neuroscience Institute at UTHSC, Memphis, TN, USA.

### 2.2. Preparation and Formulation Optimization of LCLA (Untargeted Long Circulating Liposomal AD 198)

The liposomes were prepared by the Bangham method [47] followed by extrusion via polycarbonate membranes [12,14,48]. Briefly, the lipids and drug (AD 198 free base) were weighed accurately and dissolved in 3 mL 9:1 solvent mixture of chloroform:methanol in a round bottom flask. A thin lipid film was formed at the bottom of the flask by evaporating the solvent using a BUCHI Rotavapor^®^. Rotations were maintained at rotation speed no 3 and temperature was maintained at 40 °C using a BUCHI heating bath. This step was carried out for 1 h following which the vacuum was released, and the water bath heated to 65 °C. Simultaneously, 1× PBS was prepared from the 10× PBS and added to the thin lipid film for hydration. Rotations were maintained at the number 3 setting. Hydration was carried out for 1 h which gave MLVs (multi-lamellar vesicles). The MLVs were extruded through polycarbonate filters in two steps to give SUVs (small unilamellar vesicles). Extrusion was carried out using LIPEX^®^ Extruders purchased from Northern Lipids, Burnaby, BC, Canada, connected to a high pressure ultrapure nitrogen tank. In the first step of extrusion, polycarbonate membranes of two sizes, 100 nm (nanometers) and 200 nm, were stacked and the drug loaded liposomes extruded only once using 450 psi pressure. In the second step, the resulting liposomes were extruded three times via 80 nm and 100 nm stacked membranes again using 450 psi pressure. Since there was some loss of volume during the rehydration and extrusion process, the final volume was made up to 3 mL with 1× PBS. The size and ζ-potential (zeta potential) of the final liposomes was measured using a Malvern Zetasizer Nano ZS.

### 2.3. Removal of Un-Encapsulated AD 198 

The unencapsulated drug was removed using Sephadex—G50 prefilled macro-column [49,50]. Briefly, the powdered G50 gel was rehydrated using 1× PBS for 15 min and centrifuged using a Thermo Scientific IEC CL31R centrifuge at 4 °C for 4 min at 1500 rpm. The resulting gel was washed three times using 150 µL of blank liposomes containing no AD 198, under the same centrifugation conditions as mentioned above. This was to block any non-specific retention of drug loaded liposomes in the column. Then 150 µL of the AD 198 loaded liposomes were passed through the treated column. The final eluate was reconstituted to 150 µL.

### 2.4. Analysis of Liposomal Encapsulated Drug Content 

Encapsulated AD 198 content in the liposomes was calculated using a Waters Alliance e2695 HPLC coupled to a Waters 2998 UV Photodiode Array Detector. Samples were prepared at a dilution factor of 20. Briefly, 50 µL of the purified liposomes were dissolved in 950 µL of 1:1 methanol:ethanol. Samples were briefly vortexed to give a clear solution and 300 µL transferred to Waters total recovery HPLC vials. These vials were loaded into the autosampler of the HPLC separations module. Conditions for HPLC analysis were adapted from previously optimized methods [51]. The column used for separation was Waters Nova-Pak^®^ C18 4 µm, 3.9 × 150 mm and was maintained at 30 °C throughout the separation process. The mobile phase was a 70:30 acetonitrile:pH 4.0 ammonium formate buffer. The ammonium formate buffer was prepared by adding 3.85 mL of ammonium hydroxide to 950 mL of HPLC grade water. The pH was adjusted to 4.0 using 80% formic acid and the volume was made up to 1 L. The flow rate for the mobile phase was maintained at 1.2 mL/ min, the injection volume was 20 µL and the run time for each injection was 7 min. AD 198 eluted between 3 and 4 min and was detected at a wavelength (λ) of 254 nm.

### 2.5. Determination of Phospholipid Concentration 

To calculate the amount of HSPC retained in the final formulation of LCLA, total phospholipids were estimated using a procedure adapted from Stewart et al. [52]. 2.703 g (grams) ferric chloride hexahydrate and 3.04 g ammonium thiocyanate was dissolved in 100 mL distilled water and mixed to give ferrithiocyanate reagent. To determine the concentration of HSPC in the LCLA dispersion, an HSPC standard curve was made ranging from 10 to 60 µg/mL. Analysis of these standards was done as follows. A mixture of 2 mL chloroform, 2 mL ferrithiocyanate reagent and 100 µL of the standard solution was made for each standard and vortexed vigorously for exactly one min each. The mixture was allowed to settle and the lower layer containing chloroform was aspirated carefully and transferred to a 1 mL quartz cuvette. The absorbance for each standard was measured at λ 488 against a chloroform blank. Samples of LCLA were prepared in the same method and the absorbance measured. Absorbance of the standard vs. HSPC concentration was plotted for each standard concentration and the unknown amount of HSPC in the LCLA sample was determined. 

### 2.6. LCLA AD 198 Release Study

Drug release in 1× PBS at pH 7.4 and 37 °C was tested for the LCLA’s as described by Zhang et al. [53]. Briefly, 100 µL of the LCLA’s were placed in Slide-A-Lyzer^®^ MINI Dialysis Devices, 3.5 K MWCO, 0.5 mL capacity [54,55]. 5 time points were tested; 6, 12, 24, 48 and 72 h. Each sample was tested in triplicates. The sample loaded dialysis devices were loaded into floats introduced into a 3000 mL beaker containing 3000 mL of 1× PBS preheated to 37 °C and dissolution started. Samples were taken out at the pre-determined time points and the drug content measured by HPLC as stated earlier.

### 2.7. Fab’ (Antigen Binding Fragment) Generation from Whole Anti-CD22 Antibody 

**Figure 2:**
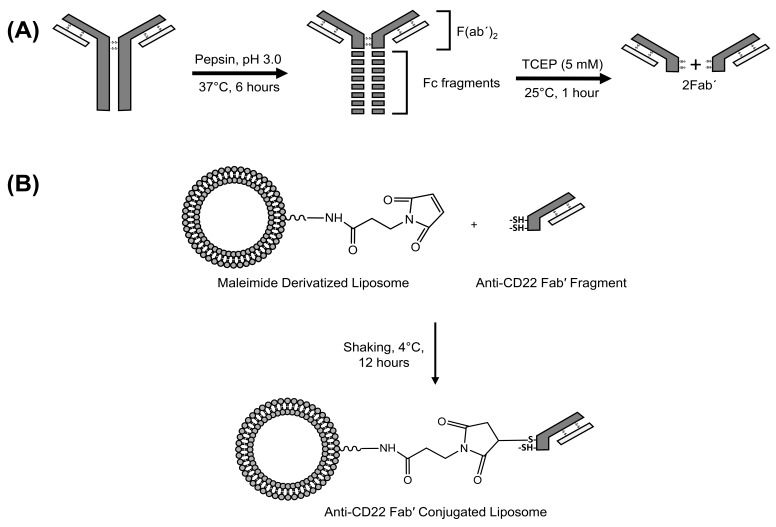
(**A**) Schematic for generation of anti-CD22 Fab’ fragments; and (**B**) Conjugation of anti-CD22 Fab’ to maleimide derivatized LCLA (untargeted long circulating liposomal AD 198).

The anti-CD22 whole antibody was first purified by passing through a G50 prefilled macro column. The Fc (constant fragment) region was first digested using immobilized pepsin. Briefly, the immobilized pepsin was first separated from the vehicle by loading the immobilized pepsin suspension into an empty macro column and centrifuging it for 2 min at 5000 *g* and 4 °C. The purified anti-CD22 monoclonal antibody was then incubated with the immobilized pepsin at pH 3.0, 37 °C for 6 h. pH 3.0 was adjusted using 1M citric acid solution. After the given time, the antibody was collected by centrifuging the immobilized pepsin and antibody digest in an empty macro spin column at 5000 *g* for 2 min at 4 °C. The collected antibody digest was then incubated with 10µl of 5 mM TCEP (tris(2-carboxyethyl)phosphine) at room temperature (RT) for 1 h. This gave 2Fab’ fragments from each molecule of antibody. The resulting digest mix was purified by filtration using two filters 100 kD and 30 kD MWCO and the appropriate fraction containing the 50 kD Fab’ was collected and used for conjugation. A schematic or this reaction is given in Figure 2A.

### 2.8. Conjugation of Fab’ to Liposomes to Give Long Circulating CD22 Targeted Liposomal AD 198 (LCCTLA)

For conjugation with antibody, liposomes were prepared by the same method as mentioned above, only 50% of m-DSPE-PEG2000 was replaced with mal-PEG2000-DSPE to serve as an anchor for the antibody. 100 µL of the Fab’ was incubated with an equal volume of the maleimide derivatized liposomes at 4 °C for 12–15 h. Following incubation, unconjugated antibody fragments were removed by gel filtration chromatography using Sepharose CL4B gel. Briefly, the 70% gel slurry in ethanol was filled in an empty PD-10 column and centrifuged at 1000 *g* for 150 s at 4 °C to remove the ethanol. Three 1× PBS washes followed the removal of ethanol. The column was the saturated with placebo liposomes in three separate runs and then the 200 µL of targeted liposomes were passed through the column. The final reaction for the conjugation between the maleimide derivatized liposomes is depicted in Figure 2B. The resulting solution was analyzed for proof of conjugation by western blotting.

### 2.9. Verification of Conjugation 

Conjugation of the 50 kD Fab’ fragment to the liposomes was verified by western blotting as done by Oliveira et al. [56]. Briefly, 4 samples were studied: the targeted liposomes, the fraction higher than 100 kD, the fraction below 50 kD and the whole antibody were quantified for total protein by the BCA assay and 20 µL (10 µL sample and 10 µL Laemmli buffer) of an equal concentration sample of protein (250 ng) were loaded into a 4–15% polyacrylamide gel. Samples were run at 100 V for approximately one hour (until the Laemmli dye reached the end of the gel). The protein bands were then transferred from the gel onto a PVDF (polyvinylidene fluoride) membrane. The membrane was probed with a mouse secondary antibody and the blot was developed on an X-ray film. 

### 2.10. Calculation of Number of Antibody Molecules per Liposome 

The number of anchors (maleimide groups) and the number of antibody molecules per liposome were calculated by first calculating the number of liposomes as previously discussed. Then number of antibody molecules and maleimide were calculated in one mL of the LCCTLA by using Avogadro’s number and substituting the values in Equations (1) to (7). 

### 2.11. Cellular Uptake of LCLA and LCCTLA by Flow Cytometry 

To determine and compare cellular uptake in CD22^+^ Daudi and CD22^−^ Jurkat cells CD22 targeted liposomes were prepared by the same method as specified previously only 0.125 mole % of the HSPC was substituted with an equal mole percent of NBD-PC for fluorescence imaging. Six time points were tested ranging from 5 min to 4 h [57]. 10 ml each of Daudi and Jurkat cells at a cell density of 7 × 10^5^ cells/mL were grown in T25 flasks for each time point. At each time point, both cell types were treated with two types of 1 µM AD 198 formulations, LCLA and LCCTLA separately. At the end of each time point the cells were centrifuged at 4 °C and 100 *g* for 4 min. The pellet obtained was washed with 1× PBS thrice and the final cell pellet was re-suspended in 1 mL of 1× PBS and tested for fluorescence intensity for NBD-PC per 10,000 cells using the BD Accuri™ C6 flow cytometer (BD Biosciences, San Jose, CA, USA). 

### 2.12. Evaluation of LCCTLA Cytotoxicity 

To determine cellular cytotoxicity, the MTT assay was used [58]. Briefly, Daudi and Jurkat cells were grown to the required cell density. The assay was set up in 96 well plates. Three different formulations of AD 198 were tested; LCCTLA, LCLA and free AD 198. Free AD 198 was prepared in DMSO such that the final concentration of DMSO was less than 3% in any treatment well. Two time points were prepared for each treatment, 24 h and 48 h. For the 24-h treatment 17,500 cells per well were plated and for the 48-h treatment 8750 cells per well were plated. Treatment was done for 10 concentrations of each of the three AD 198 formulations ranging from 0.01 µM to 3 µM and the control was cells with no drug treatment. These plates were incubated at 37 °C and 5% CO_2_ for the study time period. At the end of the study time (24 or 48 h) 15 µL of 5 mg/mL concentration of MTT dye was added to each well of study and incubated under the same conditions mentioned above for 4 more h at 37 °C. Following the 4-h incubation the insoluble formazan dye formed as a result of the reaction was dissolved in 100 µL of solubilization buffer (20% SDS in 50% DMF, 0.5% of 80% acetic acid and 0.4% 1N HCl) and incubated for 3 h at 37 °C. After incubation absorbance was read at λ 570 nm using the SpectraMax M2e^®^ microplate reader (Molecular Devices, San Jose, CA, USA).

### 2.13. Energy Dependent or Independent Pathway for LCCTLA Internalization

To confirm that the mechanism of uptake of LCCTLA particles into Daudi cells was by receptor-mediated endocytosis cellular association studies were performed [56]. Briefly, 10 mL of Daudi cells at a cell density of 7 × 10^5^ cells/mL were grown in two separate T25 flasks. One was pre-cooled to 4 °C and then treated with 1 µM fluorescent LCCTLA for one hour and the other was treated with 1 µM fluorescent LCCTLA at 37 °C for one hour. At the end of the time point, the cells were centrifuged at 4 °C and 100 *g* for 4 min. The pellet obtained was washed with 1× PBS thrice and the final cell pellet was re-suspended in 1 mL of 1× PBS and tested for fluorescence intensity for NBD-PC per 10,000 cells using the BD Accuri™ C6 Flow cytometer. 

### 2.14. Route of Uptake of LCCTLA into Daudi Cells

To determine the mechanism of entry of LCCTLA into the Daudi cells, various inhibitors were used to block specific pathways and then the uptake analyzed as done in the determination of CD22 targeted liposomal drug uptake in cells by flow cytometry. As per Douglas et al. [59] four specific inhibitors were used; amiloride for micropinocytosis, genistein or M-β-CD for caveolae and related structures mediated endocytosis and chlorpromazine for clathrin-mediated endocytosis. 10 mL Daudi cells at a concentration of 7 × 10^5^ cells/mL were incubated with the inhibitors for one hour at the following specific concentrations; amiloride 10 µM, genistein 0.2 µM and chlorpromazine 10 µg/mL. Following this the inhibitor treated cells were treated with 1 µM fluorescent CD22 targeted liposomal AD 198 and incubated for 1 h at 37 °C. At the end of the treatment the cells were processed in the same method as for the determination of LCCTLA uptake in Daudi cells by flow cytometry. 

### 2.15. Intracellular Trafficking of LCCTLA by TEM 

#### 2.15.1. MLV Preparation and Treatment of Daudi Cells 

To view the intracellular localization of LCCTLA in the Daudi cells [60,61], 15 nm sized magnetic iron oxide nanoparticles in water (with carboxylic acid functional group) were processed into the liposomes [62]. Briefly, 1% *w*/*w* iron oxide nanoparticles were added to the 1× PBS that was used to rehydrate the dried lipid film. The MLV’s thus obtained were sonicated for 5 s with 5 min intervals (extrusion was initially tried as the size reduction technique, however the liposomes failed to extrude). During the sonication and the interval, the liposomal suspension was kept in ice. The sonication was repeated three times to give magnetic LCLA (MLCLA). The anti-CD22 Fab’ was conjugated as specified previously to give MLCCTLA (magnetic LCCTLA). 10 mL Daudi cells at a density of 7 × 10^5^ cells/mL were treated with 1 µM of the MLCCTLA for 4 h. After treatment, the cells were centrifuged at 100 *g* for 5 min at 4 °C. The pellet was washed with ice cold 1× PBS thrice and suspended in 1 mL 2.5% glutaraldehyde in 1× PBS. 

#### 2.15.2. TEM Sample Preparation

Once the cells were fixed in 2.5% glutaraldehyde overnight they were spun down at 100 *g* for 5 min and washed with 10× PBS thrice for 20 min each. Then the cells were stained with a 4% osmium tetroxide in PBS solution for 1 h at RT and excess stain washed off using 10× PBS thrice for 20 min each. The cell sample was then dehydrated in an ethanol series gradually increasing from 50% ethanol to 100% ethanol in four steps. Each dehydration cycle was done once for 10 min and the 100% ethanol thrice. The cells were then infiltrated with 50% Spurr’s resin in ethanol under rotation overnight. Infiltration was continued with 100% Spurr’s resin the next day thrice for 2 h periods. With the 100% Spurr’s resin, the centrifugation was done at 800 *g* for 20 min. The cells were then embedded in fresh Spurr’s resin in a mold and cured at 65 °C for 48 h. Once the resin hardened the sample block was placed in an ultramicrotome and approximately 80 nm sections cut using a diamond knife. Chloroform was used to smoothen the sections. The sections were loaded onto copper grids and stained with uranyl acetate and lead citrate to increase contrast and electron density. The grids were then inserted into the TEM for viewing. The TEM used was the JEOL 2000EX with a high resolution digital camera and monitor (JEOL, Peabody, MA, USA). 

### 2.16. Intracellular Trafficking of LCCTLA by CLSM 

To confirm that the intracellular vesicles the magnetic liposomes were observed in were endolysosomes, cells were stained with endolysosome specific dye [63,64,65]. Briefly, 10 mL Daudi cells were cultured to a cell density of 7 × 10^5^ cells/mL and treated with 10 µL of placebo fluorescent CD22 targeted liposomes (prepared by substituting 0.125 mole % of HSPC with an equal mole % of NBD-PC, and not loading any drug) for 1 h. During the final 5 min of treatment, 50 nM LysoTracker^®^ Deep Red was added to the treated cell culture. The media was then removed by centrifugation and the cell pellet thus obtained was washed 3 times using 1× PBS. The final cell pellet was suspended in a single drop of Vectashield^®^ cell mounting medium with DAPI. This final cell suspension was mounted onto a microscope slide and covered by a Fisherbrand number 1 coverslip. The slide was allowed to dry in a fume hood for approximately 30 min and the edges were sealed using a transparent nail polish. The nail polish was also allowed to dry in a fume hood for another 30 min after which the slide was viewed under a Nikon Eclipse E800 confocal scanning laser microscope (Nikon Instruments, Melville, NY, USA). The lasers were set at the wavelength for NBD-PC, LysoTracker^®^ Deep Red and DAPI and images were obtained. 

### 2.17. Effect of LCCTLA on Cell Cycle Regulatory Molecules by Western Blot

To study the efficacy of the drug delivery system on induction of apoptosis in the cancer cells, the cells were treated with the LCCTLA and the expression of 4 proteins was monitored, Caspase 3, c-myc, p-JNK, pAKT. β-actin was used as a control to signify equal loading. The process [66] has been briefly outlined below. 

#### 2.17.1. Sample preparation

10 mL of Daudi cells at a density of 7 × 10^5^ cells/mL were treated with 1 µM of LCCTLA at three separate time points, 2, 4 and 6 h. The control was the same density and volume of cells but without any drug treatment. At the end of the treatment, cells were centrifuged in a Thermo Scientific Sorvall Legend X1 centrifuge (Thermo Fisher Scientific, Waltham, MA, USA) at 100 *g* for 4 min at 4 °C. The pellet obtained was washed with 1× PBS thrice and then suspended in 70 µL whole cell lysis buffer [Tris HCl 50 mM, NaCl (sodium chloride) 150 mM, Triton X-100 1%, SDS 0.1%, EDTA (ethylene diamine tetra acetic acid) 5 mM, Na_2_HPO_4_ (disodium phosphate) 30 mM, NaF (sodium fluoride) 50 mM, NaVO_4_ (sodium orthovandate) 0.5 mM, PMSF (phenylmethylsulfonylfluoride) 2 mM and protease inhibitor] at 1 µL/10^6^ cells. This cell suspension was sonicated using a Virtis Virsonic^®^ Ultrasonic Cell Disrupter. The samples were sonicated thrice for 5 s each with 5 min intervals. During the intervals, the cell debris suspension was placed on ice. The cell debris suspension was then centrifuged at 1000 rpm at 4 °C for 10 min and the supernatant collected. The pellet obtained was discarded.

#### 2.17.2. Polyacrylamide Gel Electrophoresis (PAGE)

The protein content in the supernatant collected was quantified using the microplate BCA (bicinchoninic acid) protein assay. A volume of protein was calculated such that all samples had an equal concentration of protein. These whole cell protein samples were then mixed with an equal volume of 2× Laemmli sample buffer and boiled for 5 min. Samples were then cooled to RT for 3 min and loaded onto a 4–15% polyacrylamide gel of 50 µL well capacity. The buffer reservoir was filled up with electrode running buffer to the given mark. The electrodes were connected to a Bio-Rad power pack and samples were electrophoresed at 100 V for approximately one hour (until the Laemmli sample dye reached near the bottom of the gel).

#### 2.17.3. Blotting

The gel was then loaded onto a transfer cassette to transfer the protein bands onto a PVDF membrane. The sized membrane was first soaked in 100% methanol followed by transfer buffer. It was then placed over the gel in the cassette and the transfer apparatus set up. A stir bar was placed at the bottom of the buffer reservoir along with a freezer pack. Transfer buffer was then poured into the reservoir to a level such that the freezer pack was fully submerged and covered the cassette completely. The electrodes were connected to a Bio-Rad power pack and the transfer was done at 100 V for one hour. 

#### 2.17.4. Primary and Secondary Antibody Probing

Once the bands were transferred to the membrane, the membrane was blocked using either 5% powdered milk or 5% BSA (bovine serum albumin) in TBST (Tris-buffered saline and Tween^®^ 20). The blocking was done at RT for 1 h on a shaker. After the blocking was complete, excess milk or BSA was washed off the membrane with TBST (a quick wash) and the primary antibody for a specific protein was added at a dilution of 1:1000 (only β-actin 1:40,000) in TBST. The blot was incubated with the primary antibody for 12–15 h at 4 °C on a rocker. At the end of the incubation period, the excess primary antibody was washed off the membrane using TBST. The wash was done thrice for 5 min each. Then, the membrane was incubated with secondary antibody specific for the particular antibody used (anti-rabbit for c-myc, pJNK, pAKT and caspase-3 and anti-mouse for β-actin) at a dilution of 1:25,000 for 1 h at RT. The excess secondary antibody was then washed off the membrane using TBST thrice for 15 min each. 

#### 2.17.5. Analysis

Once the secondary antibody was probed onto the membrane, the substrate (hydrogen peroxide + luminol) was added and briefly incubated for 1–2 min. The membrane was then loaded onto an X-ray film cassette and covered with a fresh X-ray film. This film was then developed, and the results recorded. 

#### 2.17.6. Membrane Stripping

The membrane was reused for probing another protein with another antibody. For this, the previous antibody was removed by a process called stripping and was done by a stripping buffer. The membrane already bound with the primary and secondary antibody was incubated with the stripping buffer for 5 min at 45 °C following which it was washed with TBST and was reused for probing the next protein. 

### 2.18. Statistical Methodology

All work was performed in triplicates (*n* = 3). Data are expressed as mean ± standard deviation (SD). Student’s t-test was used for data analysis; *p*-values ≤ 0.05 were considered statistically significant. 

## 3. Results

### 3.1. This Section Effect of Lipid Composition in the Bilayer

EPC and HSPC were studied for their effects on the physico-chemical properties of the liposomes such as liposomal encapsulation of AD 198, size and ζ-potential. Both lipids are derived from natural sources and are biodegradable. However, HSPC and EPC differ in their composition such that EPC is a crude mixture of saturated and unsaturated lipids of varying chain lengths, whereas HSPC is primarily composed of saturated lipids of a fixed chain length. The effect that this has on the lipid bilayer is that the longer chain length lipids in EPC increase the thickness of the bilayer (not the size of the liposome) [67]. The more saturated a lipid is, the higher is its transition temperature, thus imparting lesser fluidity and possibly increased stability [67]. A variable length between the two tail group chains also reduces the order of packing in the liposomal bilayer. Liposomes with saturated lipids such as HSPC also have higher circulation half-lives compared to liposomes with unsaturated lipids such as EPC [68,69]. 

**Figure 3:**
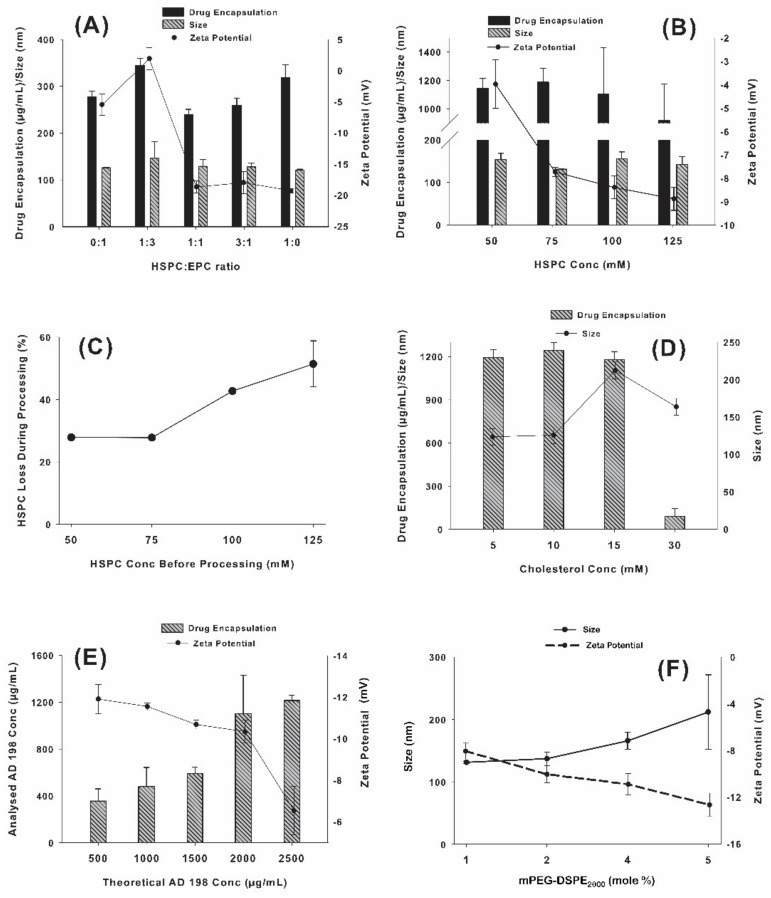
(**A**) Effect of lipid composition on AD 198 encapsulation and liposomal size; (**B**) Effect of total phospholipid concentration on liposome size, ζ-potential and AD 198 encapsulation; (**C**) Optimization of HSPC concentration; (**D**) Effect of cholesterol concentration on AD 198 encapsulation and liposome size; (**E**) Effect of AD 198 concentration on AD 198 encapsulation and ζ-potential; and (**F**) Effect of mPEG2000-DSPE concentration on ζ-potential and liposome size.

Studies were performed using HSPC and EPC at various ratios to optimize the physico-chemical properties of LCLA. Figure 3A shows EPC and HSPC formulations studied in various ratios for their effect on AD 198 encapsulation, liposomal size, and ζ-potential. It was observed that the size of the liposomes was not related to the lipid composition in any definitive manner. One hundred percent HSPC gave the smallest liposomal size. Liposomal AD 198 concentration was also not associated with the lipid composition in a significant manner with 100% HSPC giving highest AD 198 encapsulation. Dependence of ζ-potential on the lipid composition however displayed a trend compared to size and drug encapsulation. As the percentage of HSPC was increased, ζ-potential became more electronegative with an average value of −19.3 mV at 100% HSPC concentration. Although the other formulations containing some percentage of EPC produced liposomes of size, ζ-potential and AD 198 encapsulation comparable to the formulation with 100% HSPC, the latter was selected as the formulation of choice this point forward due to the difficulty of working with EPC which can be attributable to its lesser stable nature which makes for a very hygroscopic powder and lesser stable formulations due to the unsaturation in its structure.

### 3.2. Effect of Total Phospholipids on AD 198 Encapsulation, Liposomal Size and ζ-Potential

Optimum concentration of HSPC was tested by comparing liposomes with 5 different concentrations of HSPC: 25, 50, 75, 100 and 125 mM. The drug concentration was kept constant at 2000 µg/mL. The batch with 25 mM HSPC failed to extrude. This may be attributed to the excess amount of drug in the system that the little amount of carrier (HSPC) would need to encapsulate. As seen from experiments, it is speculated that the un-encapsulated drug forms a thin layer on the membrane thus preventing the liposomes from extruding. The results for 50–125 mM HSPC are shown in Figure 3B. It would be logical to believe that with increase in the amount of HSPC in the system, which is the major encapsulating material, the amount of encapsulated drug would tend to increase. However, the results suggest that the amount of drug encapsulated may not necessarily be related to the amount of lipid in the system. This result can be explained with Figure 3C which indicates that during processing, a considerable amount of phospholipid was being lost as the total lipid content was increased beyond 75 mM. As portrayed in Figure 3C it was observed that the amount of HSPC loss during processing at 75 mM HSPC is approximately 25% (almost the same as 50 mM). However, at 100 mM and 125 mM the amount of phospholipid loss was more than 40%, whereas the benefits in terms of AD 198 encapsulation and liposomal size were negligible (Figure 3B). Although there was a pronounced increase in the net negative charge on the liposomal surface, as depicted in Figure 3B, the size of the liposome increased considerably which allowed the conclusion of total phospholipid concentration at 75 mM to be optimum for development purposes. An additional justification was that the extrusion time with total phospholipid concentration higher than 75 mM increased considerably (more than 10 times). 

There was an increase in the negative charge of the liposomes when the phospholipid concentration was increased from 50 mM to 75 mM. This was followed by a reduced degree of increase from 75 mM to 100 mM and 125 mM can possibly be explained by the excess amount of AD 198 that remains un-encapsulated. Since AD 198 has a cationic nature and HSPC an anionic nature, the excess AD 198 in the dispersion could be adsorbed onto the liposomal surface thus shielding the anionic nature of HSPC. 

### 3.3. Effect of Cholesterol Concentration on AD 198 Encapsulation and Liposomal Size

Cholesterol is incorporated into the membrane to obtain an optimum bilayer fluidity [67,70]. However, with encapsulation of hydrophobic drugs in the bilayer it is important to consider effects of cholesterol concentrations on the encapsulation of the drug even before bilayer fluidity is considered. This is shown in Figure 3D which depicts the results from a study on the effects of increasing cholesterol concentrations. Four concentrations of cholesterol ranging from 5 mM to 30 mM were studied. Liposomal size was relatively similar for 5 and 10 mM cholesterol concentrations. However, the size increased considerably at 15 and 30 mM cholesterol. This may be attributed to the liposomal bilayer trying to accommodate both the AD 198 and the increasing cholesterol molecules which could possibly increase the liposomal size. AD 198 concentration reduced more than 10-fold from approximately 1000 µg/mL to 90 µg/mL, when cholesterol concentration was increased from 15 mM to 30 mM. Since part of the cholesterol molecular structure is similar to that of AD 198, the excess cholesterol molecules trying to occupy volume within the bilayer may be pushing out AD 198. This was also in agreement with the liposomal size results. Once excess AD 198 was pushed out, the liposomal size reduced by approximately 50 nm. These results suggested that in view of AD 198 encapsulation and liposomal size, 10 mM cholesterol concentration was optimum for LCLA. It should also be noted that with increasing cholesterol concentrations bilayer permeability decreases as demonstrated by Hu et al. [71], which is an advantage for long circulating liposomes.

### 3.4. Effect of AD 198 Concentration on AD 198 Encapsulation and ζ-Potential

Five concentrations of AD 198 ranging from 500 µg/mL to 2500 µg/mL were tested to determine their effect on AD 198 encapsulation and ζ-potential. Figure 3E shows that as the AD 198 concentration was increased from 500 to 1500 µg/mL the AD 198 encapsulation increased steadily. However, when the AD 198 concentration was increased from 1500 to 2000 µg/mL, the AD 198 encapsulation almost doubled. However, if the AD 198 concentration was increased any further, there was not a considerable increase in AD 198 encapsulation. The ζ-potential was relatively constant up to 2000 µg/mL. However, once the AD 198 concentration reached 2500 µg/mL, the electronegativity of the particles reduced. Again, this can be attributed to the excess un-encapsulated cationic AD 198 adsorbing onto the predominantly anionic HSPC liposomes, which will shield the negative charge due to HSPC. Hence, 2000 µg/mL was selected as the optimum AD 198 concentration and hereon this was the concentration used for further studies.

### 3.5. Optimization of mPEG2000-DSPE Concentration

To determine the optimum amount of mPEG2000-DSPE, four concentrations of mPEG2000-DSPE ranging from 1 to 5 mole % were studied. Figure 3F represents the effect of mPEG2000-DSPE concentration on liposomal size and ζ-potential. The liposomal size was observed to increase with increasing concentrations of mPEG2000-DSPE. Conversely, ζ-potential was observed to turn more electronegative with an increase in mPEG2000-DSPE concentration which may be attributable to the anionic nature of mPEG2000-DSPE. mPEG2000-DSPE is a large molecule (MW = 2805.497). The increase in the number of mPEG2000-DSPE molecules may cause the net size of the liposome to increase as justified by Woodle and co-workers [72]. However, the 2 mole % mPEG2000-DSPE imparted optimum size and ζ-potential to the formulation and this was selected for further studies. 

### 3.6. LCLA Drug Release

**Figure 4:**
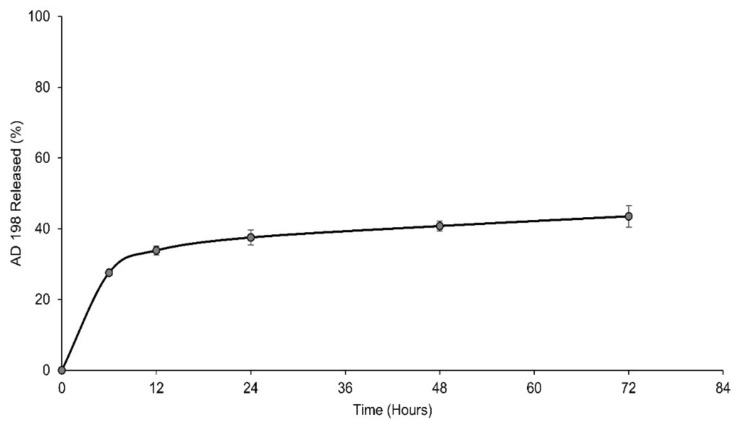
AD 198 release profile from LCLA at 37 °C.

LCLA was tested for its release characteristics in 1× PBS at 37°C. Figure 4 shows that approximately 30% AD 198 release was observed over the first 12 h following which only about 10% more AD 198 was released over the next 48 h. This may be due a biphasic release mechanism in which the drug is released in two separate phases. Initially the drug was released in a burst mode from drug simply adhered to the liposomal surface, followed by the release of drug from the liposomal bilayer. Possibly there may be micelles formed from the monomer lipid molecules which were not incorporated into liposomes. If these monomer lipids are above their critical micelle concentration (CMC), they will form micelles. Micelles are less stable compared to liposomes and may release drug faster than liposomes. Once micelle drug release is over the liposomal AD 198 release predominantly determines the kinetics. The release pattern is necessary with the theory of active targeting in which the liposome requires a certain period of time before it encounters a CD22^+^ malignant B cell, binds to it and is internalized. The drug must largely be released once the liposome is inside the malignant cell. Therefore, the delayed release of AD 198 over >72 h is beneficial for the LCLA drug delivery system.

### 3.7. Number of AD 198 Molecules/Liposome

The number of average HSPC molecules per mL of the LCLA was calculated by using the Avogadro’s number, 6.023 × 10^23^ molecules/mole. This gave an average of 3.25 × 10^22^ HSPC molecules/mL of LCLA as per the analyzed concentration of HSPC, 54 mM from the HSPC assay results. The number of average AD 198 molecules/mL of LCLA were calculated similarly from the analyzed concentration of AD 198 to be 1250 µg/mL. This gave an average of 1.05 × 10^21^ AD 198 molecules/mL of LCLA. The outer surface area of the liposomal bilayer was calculated from Equation (1).

(1) $$\mathrm{Outer}\;\mathrm{Surface}\;\mathrm{Area}=4\pi r^{2}$$

Here ‘*r*’ was the average outer radius of the liposome. The thickness of the bilayer was denoted ‘*h*’ and is 5 nm [73]. Then the inner surface area was calculated from Equation (2).

(2) $$\mathrm{Inner}\;\mathrm{Surface}\;\mathrm{Area}=4\pi {\left(r-h\right)}^{2}$$

The cross-sectional area of a phosphatidylcholine headgroup was denoted as ‘*a*’ with a value of 0.71 nm^2^ [74] and the number of HSPC molecules per liposome calculated from Equation (3).

(3) $$\mathrm{Number}\;\mathrm{of}\;\mathrm{HSPC}\;\mathrm{molecules}/\mathrm{liposome}=\frac{4\pi \left[r^{2}+{\left(r-h\right)}^{2}\right]}{a}$$

$$\mathrm{Number}\;\mathrm{of}\;\mathrm{HSPC}\;\mathrm{molecules}/\mathrm{liposome}=\frac{17.69\left[60^{2}+{\left(60-5\right)}^{2}\right]}{0.71}=117196$$

This gave us the number of liposomes/mL from Equation (4).

(4) $$\mathrm{Number}\;\mathrm{liposomes}/\mathrm{mL}=\frac{\mathrm{Number}\;\mathrm{of}\;\mathrm{HSPC}\;\mathrm{molecules}/\mathrm{mL}}{\mathrm{Number}\;\mathrm{of}\;\mathrm{HSPC}\;\mathrm{molecules}/\mathrm{liposome}}$$

$$\mathrm{Number}\;\mathrm{liposomes}/\mathrm{mL}=\frac{3.25\times 10^{22}}{117196}\;=2.77\times 10^{17}$$

Number of average AD 198 molecules per liposome were calculated from Equation (5).

(5) $$\mathrm{Number}\;\mathrm{of}\;\mathrm{AD}\;198\;\mathrm{molecules}/\mathrm{liposome}=\frac{\mathrm{Number}\;\mathrm{of}\;\mathrm{AD}\;198\;\mathrm{molecules}/\mathrm{mL}}{\mathrm{Number}\;\mathrm{of}\;\mathrm{liposomes}/\mathrm{mL}}$$

$$\mathrm{Number}\;\mathrm{of}\;\mathrm{AD}\;198\;\mathrm{molecules}/\mathrm{liposome}=\frac{1.05\times 10^{21}}{2.77\times 10^{17}}=3790$$

### 3.8. Verification of Anti-CD22 Fab’ Conjugation

**Figure 5:**
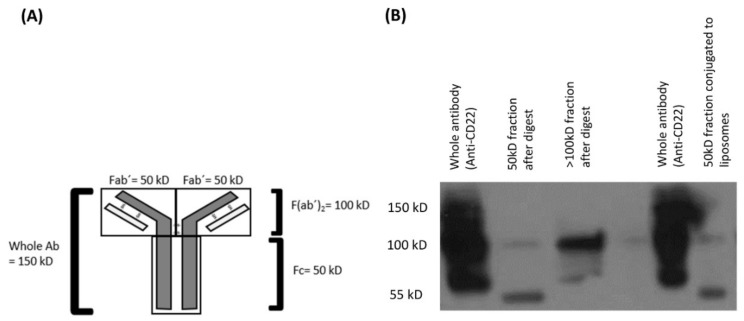
(**A**) Verification of anti-CD22 Fab’ conjugation; (**B**) Verification of conjugation of anti-CD22 Fab’ by western blotting.

A western blot of the LCCTLA was run with pure antibody digest fractions and the whole antibody. The results are shown in Figure 5. The whole antibody gave a very intense band at the 150 kD region whereas the concentration of protein for the fraction below 50 kD was very low and bands relatively faint. Figure 5A shows the possible combinations of the antibody digests that may be produced and may show up on the blot. Fab’ would be 50 kD each, F(ab’)_2_ would be 100 kD, the Fc region has been digested by pepsin thus would be broken into very small peptides possible smaller than 10 kD, and undigested antibody would be 150 kD. 

In Figure 5B in the first three lanes are for pure antibody and digests. Lane 1 is undigested whole antibody, lane 2 is the digested Fab’ fragment and lane 3 is the fraction above 100 kD. The last lane is LCCTLA and here we observed a clear band at approximately 50 kD. Since we had removed other fragments such as the Fc pepsin digests and whole antibody from the 50 to 100 kD fraction, this was possibly the band for the anti-CD22 Fab’. The whole antibody is shown beside the LCCTLA lane for reference purposes. The appearance of a strong band at 50 kD in the LCCTLA sample proved that conjugation between the liposomes and the Fab’ was successful.

### 3.9. Number of Anti-CD22 Fab’ and Maleimide per LCCTLA

2.2 mg/mL of mal-PEG2000-DSPE was added to make LCCTLA particles. This equals 748 µM of mal-PEG2000-DSPE. Using Avogadro’s number, we got an average of 4.5 × 10^20^ mal-PEG2000-DSPE molecules/mL. As specified in the method, the number of maleimide’s per liposome can be calculated by Equation (6).

(6) $$\mathrm{No}.\;\mathrm{of}\;\mathrm{maleimide}\;\mathrm{molecules}/\mathrm{liposome}=\frac{\mathrm{No}.\;\mathrm{of}\;\mathrm{maleimide}\;\mathrm{molecules}/\mathrm{mL}}{\mathrm{No}.\;\mathrm{of}\;\mathrm{liposomes}/\mathrm{mL}}$$

$$\mathrm{Number}\;\mathrm{of}\;\mathrm{maleimide}\;\mathrm{molecules}/\mathrm{liposome}=\frac{4.5\;\times \;10^{20}}{2.77\times 10^{17}}\approx 1626$$

The average number of anti-CD22 Fab’ molecules were calculated in a similar method. The analyzed concentration of Fab’ fragments in LCCTLA was 313 µg/mL which equals 6.26 µM. Using Avogadro’s number, the number of anti-CD22 Fab’ molecules/mL were calculated to be 3.77 × 10^18^ molecules/mL. Substituting these numbers into Equation (7):

(7) $$\mathrm{Number}\;\mathrm{of}\;\mathrm{Fab}’/\mathrm{liposome}=\frac{\mathrm{Number}\;\mathrm{of}\;\mathrm{Fab}’/\mathrm{mL}}{\mathrm{Number}\;\mathrm{of}\;\mathrm{liposomes}/\mathrm{mL}}$$

$$\mathrm{Number}\;\mathrm{of}\;\mathrm{Fab}’/\mathrm{liposome}=\frac{3.77\times 10^{18}}{2.77\times 10^{17}}\approx 13\;\mathrm{anti}\text{­}\mathrm{CD}22\;\mathrm{Fab}’/\mathrm{liposome}$$

The physicochemical properties of the liposomes after anti-CD22 Fab’ conjugation was as follows: mean size was 148.6 ± 4 nm, mean ζ-potential was −10.7 ± 2 mV and average drug encapsulation was about 400 µg/mL.

### 3.10. Cellular Uptake of LCLA and LCCTLA 

**Figure 6:**
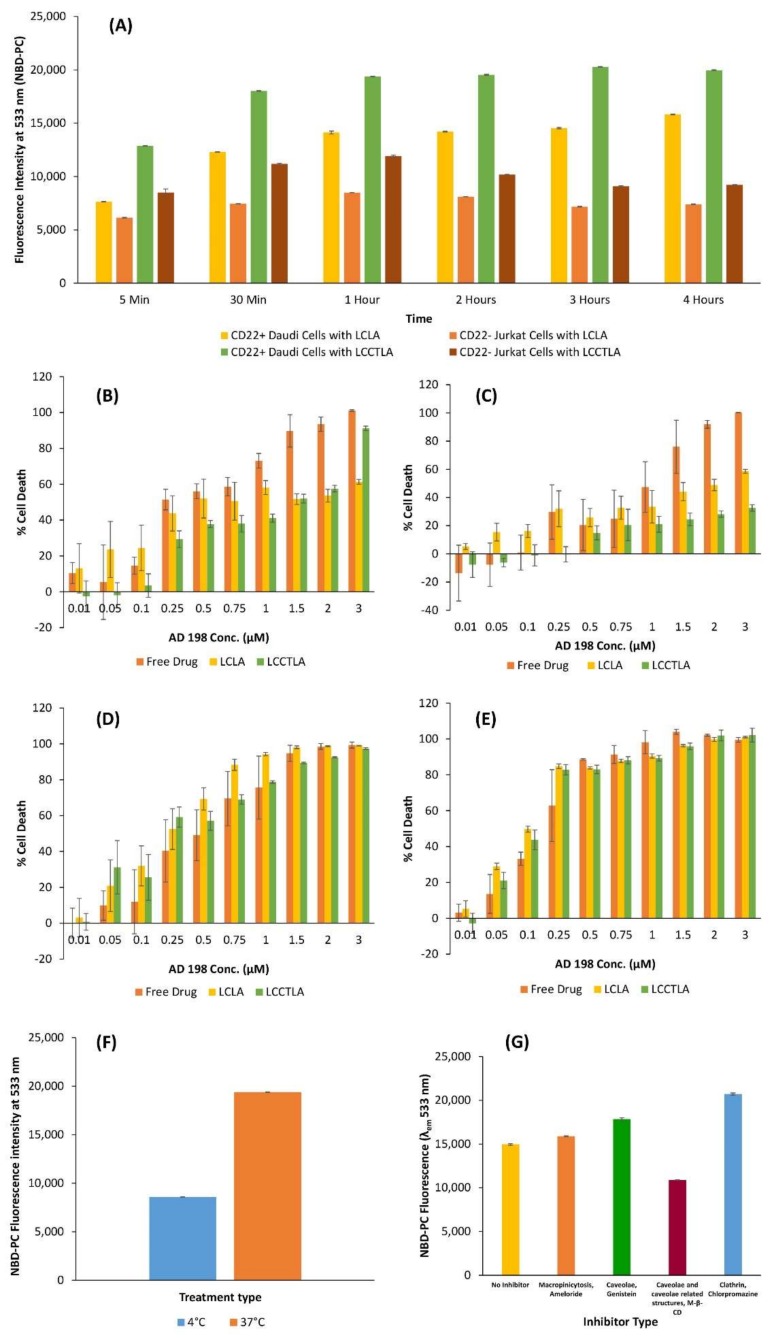
(**A**) Time-dependent cell uptake of LCLA and LCCTLA (long circulating CD22 targeted liposomal AD 198) in Daudi and Jurkat cells; (**B**) 24 h Daudi AD 198 cytotoxicity; (**C**) 24 h Jurkat AD 198 cytotoxicity; (**D**) 48 h Jurkat cytotoxicity; (**E**) 48 h Daudi cytotoxicity; (**F**) Cell association of LCCTLA in Daudi cells; and (**G**) LCCTLA uptake in Daudi cells under different inhibitors.

Flow cytometry and confocal laser scanning microscopy (CLSM) were utilized to determine the uptake of LCLA and LCCTLA in CD22 expressing Daudi and CD22 non-expressing Jurkat cells. Figure 6A summarizes the results for cellular uptake of both formulations in both Daudi and Jurkat cells. It was observed that the maximum uptake at each time point was for LCCTLA in Daudi cells. The least uptake was seen with LCLA treated Jurkat cells at every time point. Daudi cells treated with LCLA and Jurkat cells treated with LCCTLA had intermediate uptake. Maximum uptake in Daudi cells treated with LCCTLA was understandable due to the CD22 receptor being overexpressed on the Daudi cells and the LCCTLA having the antibody for this overexpressed receptor. However, the Jurkat cells having higher uptake with LCCTLA than LCLA is more complicated to explain. One theory we suggest is that the Jurkat cells may have some receptor on their surface with which the anti-CD22 Fab’ non-specifically interacts, thus causing higher uptake. Also, it was seen for Daudi cells treated with LCCTLA, the uptake plateaued at approximately one hour. Therefore, maximum uptake had already taken place by one hour.

### 3.11. Analysis of Cytotoxicity of LCCTLA 

Three formulations of AD 198 (free AD 198, LCLA and LCCTLA) were tested in Daudi and Jurkat cells. The study was performed to test the cytotoxic effect of the formulations at two lengths of exposure, 24 and 48 h. Figure 6B to 6E depict the results of this study. Figure 6B exhibits the cytotoxic effects of the three formulations, free drug, LCLA and LCCTLA over 24 h in Daudi cells. The difference in cytotoxicity between different formulations is clearly pronounced. From 0.01 µM to 3 µM AD 198 concentration, free AD 198 is most cytotoxic, with an IC_50_ of approximately 0.25 µM, as shown in Table 1 below. LCLA has an IC_50_ at about 0.5 µM, but LCCTLA have an IC_50_ of about 1.5 µM. Up to 1 µM LCLA was more cytotoxic than LCCTLA and almost equally cytotoxic at 1.5 µM. At 2 µM and 3 µM, LCCTLA displayed higher cytotoxicity. This may be attributable to the process of receptor-mediated endocytosis which may possibly require additional time to endocytose the targeted liposomes. The Jurkat cells are devoid of the CD22 receptors and thus we observed the results as displayed in Figure 6C, which are the results of the 24-h study in Jurkat cells. Since the CD22 receptor is absent in Jurkat cells, LCCTLA was not as cytotoxic as LCLA or free AD 198. 

In the 48-h study, a steadily increasing cell death pattern was noticed in both Jurkat (Figure 6D) and Daudi cells (Figure 6E) from 0.01 µM to 0.5 µM for all formulations. Then from 0.75 µM up to 3 µM, there is not much of a pronounced difference between concentrations and between the formulations. This result may be attributed to the overburdening of the cells with the drug in all types of formulations. Since the drug is not being cleared as in in vivo systems, all the drug from the formulation eventually enters the cells and kills them. This may be controlled by developing a system that would mimic blood circulation as in whole animals. One such model was developed by Budha et al. at the University of Tennessee Health Science Center [75]. Nevertheless, in vivo models would give more accurate representations of how the drug would behave in clinical settings. 

### 3.12. Cellular Association

Figure 6F shows the results of the cellular association studies. It was observed that the LCCTLA uptake in Daudi cells was significantly reduced when the uptake study was being performed at 4 °C compared to when it was done at 37 °C. Receptor-mediated endocytosis is a specific process that requires ATP for appropriate functioning and is also temperature dependent (with optimum temperature being 37 °C) [76]. It may be possible that the reduced uptake in the 4 °C study group was due to the uptake mechanism for the LCCTLA being receptor-mediated endocytosis. Since the mechanism is greatly reduced and even possible halted, the uptake seen may be a result of the receptor associated LCCTLA being internalized before the washes were done. This result suggests that the mechanism of uptake of LCCTLA into CD22 expressing Daudi cells is receptor-mediated endocytosis.

### 3.13. LCCTLA Particles Are Endocytosed into Cells by a Clathrin- and Caveolae-Independent Pathway

Figure 6G shows the results for LCCTLA uptake under the effect of inhibitors for certain specific pathways. The control data is for uptake results under no inhibitor use. Compared to the control, the only pathway that was significantly inhibited was caveolae and caveolae related structures. All other pathways, macropinocytosis, caveolae-mediated internalization and clathrin-mediated internalization, LCCTLA uptake seems to have somewhat increased significantly. The results are not clear as to why the uptake is increasing in most pathways under inhibitor use and decreasing in one (similar to caveolae mediated uptake, in which the uptake increased). It is possible that none of these pathways is the mechanism for uptake of LCCTLA in CD22 expressing Daudi cells, which suggests that LCCTLA uptake could be via the fourth pathway which is independent of clathrin and caveolin proteins and that the uptake of LCCTLA was not affected in the presence of these inhibitors because its uptake was not dependent on those pathways [77]. This complete mechanism of this pathway is not yet fully understood especially during the late stage of vesicle formation. 

### 3.14. LCCTLA Nanoparticles Were Localized Intracellularly in Endosomes

**Figure 7:**
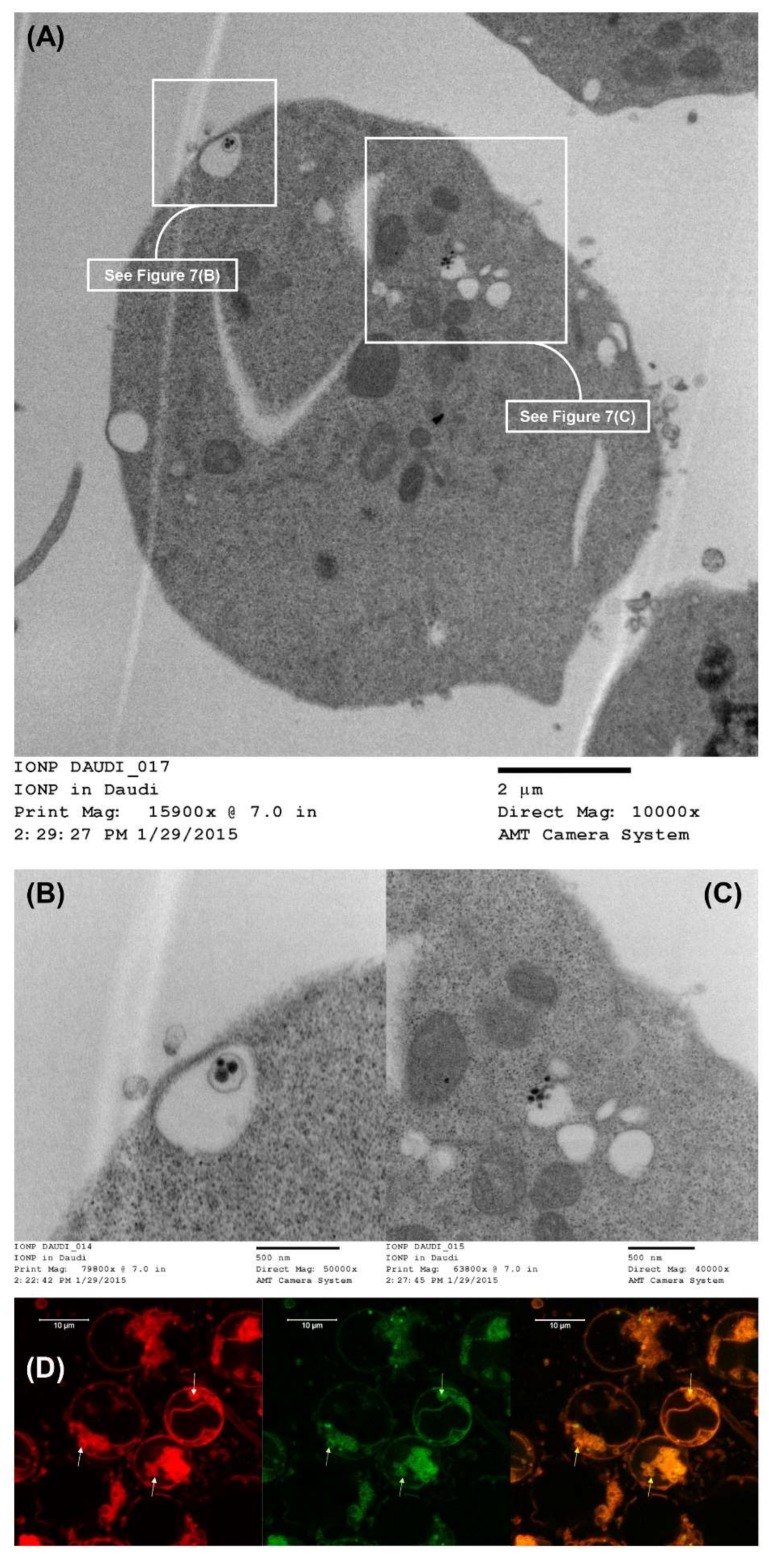
(**A**) TEM image of LCCTLA intracellular localization in Daudi cells; (**B**) Enlarged from Figure 7A showing an MLV of MLCCTLA inside an endosomal structure; (**C**) Enlarged from Figure 7A showing SUV’s of MLCCTLA inside an endosomal structure; and (**D**) CLSM image of intracellular localization of LCCTLA in endolysosomes.

Figure 7A shows a whole Daudi cell image acquired by TEM post-MLCCTLA treatment. Two parts of the image have been enlarged in Figure 7B,C. As in Figure 7B an MLV inside an endocytotic vesicle can be observed. The size of each of the three dark structures inside the vesicle are approximately 70 nm in diameter, indicating that these are the SUV’s inside the outer, larger MLV. In Figure 7C another endocytotic vesicle carrying SUV’s was observed. These SUV’s are approximately 50 nm in diameter. The size of these vesicles corresponds with the size of the MLCCTLA (data not shown here). This data confirms that the intracellular localization of the MLCCTLA is in endosomes. Later, these endosomes must fuse with lysosomes to form endolysosomes to release AD 198 [78].

### 3.15. The Endosomes Fuse with Lysosomes to Give Endolysosomes

Figure 7D shows the images for the study to determine if the endosome in which the LCCTLA were localized were endolysosomes. The cells shown here are Daudi cells treated with 10 µL of placebo LCCTLA and LysoTracker^®^ Deep Red (a dye that binds to late endolysosomes). After washing off the excess placebo LCCTLA, the images showed red circular structures inside the Daudi cells (white arrows). Since the dye for tagging the endolysosomes was red, it was deduced that these circular red structures were the endolysosomes. The liposomes were processed with NBD-PC, a fluorescent green lipid, which also show up in the images as bright green (green arrows). When these two images were overlaid, it was observed that the endolysosomes and the LCCTLA were co-localized inside the cells (yellow arrows). This establishes that the LCCTLA were intracellularly localized in endosomes (as from Figure 7B,C) and these endosomes were later fusing with the lysosomes to give endolysosomes (Figure 7D). 

### 3.16. LCCTLA Activates Classical Apoptotic Pathways

**Figure 8:**
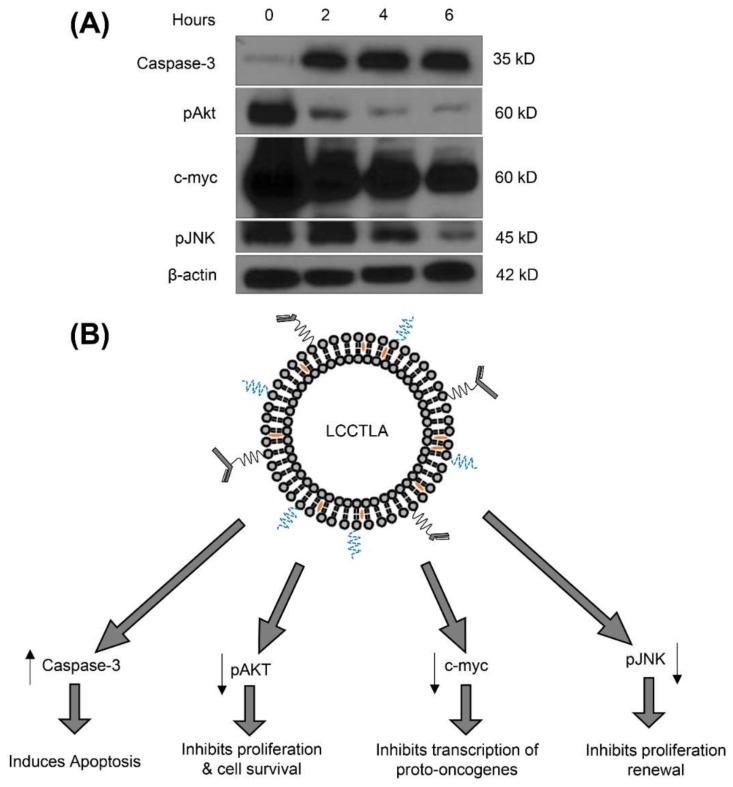
(**A**) Western blot results for the expression patterns post LCCTLA treatment for 3 time points, 2, 4 and 6 h; and (**B**) Effect of LCCTLA on the expression of caspase-3, pAKT, c-myc and pJNK.

With LCCTLA treatment in Daudi cells, it was observed that expression of oncogenic markers decreased, and apoptotic markers increased. Figure 8B shows the proteins that were affected along with their functions and Figure 8A gives a comparison of their expressions with the control. C-myc is a marker for Burkitt’s lymphoma and is a regulator gene that codes for a transcription factor [79]. The protein is multi-functional playing roles in apoptotic inhibition and cell cycle progression. Treatment with LCCTLA reduced the expression of c-myc in a time-dependent manner as shown in Figure 8A. Similar results were also demonstrated by Edwards et al. [45]. 

LCCTLA, once internalized is believed to release drug due to pH reduction in the endolysosomes. The intracellularly released AD 198 binds to PKC holoenzyme. Later the catalytic segment (CS) dissociates. The CS activates PLS3 which depolarizes the mitochondria releasing Cyt C. The Cyt C activates caspase 3. Caspase 3 being an apoptotic protein eventually results in apoptosis [40]. Thus, we observed a time-dependent increase in the concentration of caspase 3 as depicted in Figure 8A, which is a hallmark of cell death via apoptosis. There is a possibility for AD 198 to activate caspase-3 by other pathways but these are not well understood yet. 

Protein kinase B also known as Akt is a serine-threonine specific protein kinase which plays a key role in multiple process in the cell such as cell proliferation, transcription, and apoptosis. It is capable of initiating cell survival via growth factor dependent and independent pathways [80]. Phosphorylated Akt is the activated form of Akt which is necessary for it to activate or deactivate its substrates [81,82]. In Figure 8A, we observed the expression of pAKT decreasing in a time-dependent manner compared to the control, which means that LCCTLA also inhibited cell proliferation via suppression of pAKT. JNK’s or c-Jun N-terminal kinases are master protein kinases that regulate many processes in the cell such as inflammation, cell proliferation and differentiation and apoptosis. They belong to the mitogen-activated protein kinase family. Their active role in cancer development is now well-established [83]. JNK is activated by phosphorylation (pJNK). pJNK in turn phosphorylates multiple protein depending on its isoform. Figure 8A shows a time-dependent suppression of pJNK by treatment of Daudi cells with LCCTLA, thus proving that cell proliferation is also inhibited by suppression of pJNK. 

## 4. Summary and Conclusions

A prototype formulation of AD 198 loaded liposomes (LCLA) was developed that would be able to encapsulate maximum AD 198 and have optimum parameters for effective delivery of the encapsulated drug. The optimized composition of LCLA was as follows. HSPC was the lipid of choice and was used at 75 mole %, mPEG2000-DSPE 2 mole %, cholesterol 10 mole % and AD 198 2 mg/mL. The physicochemical parameters of the optimized formulation were as follows; size 115–145 nm, ζ-potential −8 to −15 mV, AD 198 encapsulation 1000–1500 µg/mL and dissolution of not more than 30% AD 198 occurred for 72 h. The size of the LCLA as per TEM was found be in the range of 80–90 nm. TEM micrographs indicated a roughly spherical morphology of the liposomes. It was calculated that the number of HSPC molecules per liposome were approximately 117,196 and the number of AD 198 molecules were approximately 3790 per liposome. 

To achieve active targeting, it was necessary to conjugate a ligand for a specific receptor on the surface of the malignant B cells. For this purpose, CD22 was selected as the receptor to be targeted on the malignant B cells. CD22 was selected due to its property of receptor mediated endocytosis upon interaction with the ligand and because it was overexpressed in malignant B cells. The anti-CD22 monoclonal antibody, RFB4, was selected as the targeting ligand. Since earlier studies by other research groups had proven that the circulating half-life of targeted liposomal systems was higher if targeted using just the Fab’ conjugated to the liposomes rather than the whole antibody, only the anti-CD22 Fab’ conjugation was optimized. Numerous methods for conjugating a ligand to the liposome exist. The one selected for conjugating the anti-CD22 Fab’ to the LCLA was with the thioether bond. The reason for selecting this strategy was its minimum use of harsh reagents and provision of a strong covalent chemical bond ensuring the stability of the targeted liposomal system. Proof of conjugation by the thioether bonding was provided by a western blot of the targeted liposomes which evidently portrayed a band at the 50 kD region which was the molecular weight of the anti-CD22 Fab’. It was calculated that every liposome displayed an average of approximately 13 anti-CD22 Fab’ molecules.

Whether or not the 13 anti-CD22 Fab’ molecules were sufficient to effectively target and deliver the liposomal AD 198 to the malignant B cells was determined by testing the long-circulating CD22 targeted liposomal AD 198 (LCCTLA), in vitro in CD22 overexpressing Daudi cells and comparing this result with non-CD22 expressing Jurkat cells. It was seen that Daudi cells had a significantly higher uptake of the LCCTLA compared to Jurkat cells, which confirmed specificity of the delivery system. The MTT assay results for cell cytotoxicity suggested a delay in cell kill for Daudi cells treated with LCCTLA, but this could be explained by the method of endocytosis that they underwent which would take more time compared to the diffusion mechanism for LCLA and solution AD 198. Nevertheless, cytotoxicity by the LCCTLA in Daudi cells was highest for a 24-h study. However, the results from the 48-h study suggest that studies in animals need to be performed in which unbound drug would be cleared faster, and this would mimic clinical settings better. 

The functioning of LCCTLA was explained by the several studies performed in vitro. Cellular association studies determined that the endocytotic mechanism was an energy dependent mechanism and it was further ascertained that the mechanisms of endocytosis possibly were a clathrin- and caveolin-independent pathway. This pathway has not been fully understood yet particularly the later stages of internalization. However, it was successfully determined that after endocytosis, the liposomes were localized in endolysosomes. This result suggested that the drug release would take place due to liposomal breakdown by the low lysosomal pH. Once the drug was released into the cytosol, it functioned via the activation of apoptotic proteins such as caspase-3 and the suppression of oncoproteins such as c-myc. These verifications were deduced by protein expression studies performed in Daudi cells post-LCCTLA treatment. 

In conclusion, targeted drug delivery with AD 198 was more potent and specific compared to other untargeted formulations. Further studies in small animal models are necessary to ascertain the efficacy of the system in more clinically relevant models. 

## Figures and Tables

**Table 1:** IC50 values for the formulation in Daudi and Jurkat cells.

Study Duration	24 h			48 h		
Formulation Type	Free Drug	LCLA	LCCTLA	Free Drug	LCLA	LCCTLA
**Cancer Cell Type**						
**Daudi**	0.243	0.481	1.443	0.199	0.101	0.114
**Jurkat**	1.054	2.046	4.608*	0.509	0.238	0.438

LCLA: Untargeted long circulating liposomal AD 198; LCCTLA: Long circulating CD22 targeted liposomal AD 198.

## References

[1] Barenholz Y.C. (2012). Doxil^®^—The first FDA-approved nano-drug: Lessons learned. J. Control. Release.

[2] Venditto V.J., Szoka F.C. (2013). Cancer nanomedicines: So many papers and so few drugs!. Adv. Drug Deliv. Rev..

[3] Green M.R., Manikhas G.M., Orlov S., Afanasyev B., Makhson A.M., Bhar P., Hawkins M.J. (2006). Abraxane, a novel cremophor-free, albumin-bound particle form of paclitaxel for the treatment of advanced non-small-cell lung cancer. Ann. Oncol..

[4] Petre C.E., Dittmer D.P. (2007). Liposomal daunorubicin as treatment for kaposi’s sarcoma. Int. J. Nanomed..

[5] Deitcher O.R., O’Brien S., Deitcher S.R., Thomas D.A., Kantarjian H.M. (2011). Single-agent vincristine sulfate liposomes injection (marqibo^®^) compared to historical single-agent therapy for adults with advanced, relapsed and/or refractory philadelphia chromosome negative acute lymphoblastic leukemia. Blood.

[6] Rodriguez M., Pytlik R., Kozak T., Chhanabhai M., Gascoyne R., Lu B., Deitcher S.R., Winter J.N. (2009). Vincristine sulfate liposomes injection (marqibo) in heavily pretreated patients with refractory aggressive non-hodgkin lymphoma. Cancer.

[7] Bharali D.J., Mousa S.A. (2010). Emerging nanomedicines for early cancer detection and improved treatment: Current perspective and future promise. Pharmacol. Ther..

[8] Davis M.E., Chen Z.G., Shin D.M. (2008). Nanoparticle therapeutics: An emerging treatment modality for cancer. Nat. Rev. Drug Discov..

[9] Torchilin V.P. (1994). Immunoliposomes and pegylated immunoliposomes: Possible use for targeted delivery of imaging agents. ImmunoMethods.

[10] Allen T.M. (2002). Ligand-targeted therapeutics in anticancer therapy. Nat. Rev. Cancer.

[11] Allen T.M., Mumbengegwi D.R., Charrois G.J.R. (2005). Anti-CD19-targeted liposomal doxorubicin improves the therapeutic efficacy in murine b-cell lymphoma and ameliorates the toxicity of liposomes with varying drug release rates. Clin. Cancer Res..

[12] Allen T.M., Sapra P., Moase E. (2002). Use of the post-insertion method for the formation of ligand-coupled liposomes. Cell. Mol. Biol. Lett..

[13] Cheng W.W., Allen T.M. (2008). Targeted delivery of anti-CD19 liposomal doxorubicin in b-cell lymphoma: A comparison of whole monoclonal antibody, fab’ fragments and single chain fv. J. Control. Release.

[14] De Menezes D.E.L., Kirchmeier M.J., Gagne J.F., Pilarski L.M., Allen T.M. (1999). Cellular trafficking and cytotoxicity of anti-CD19-targeted liposomal doxorubicin in b lymphoma cells. J. Liposome Res..

[15] Pillai G. (2014). Nanomedicines for cancer therapy: An update of fda approved and those under various stages of development. SOJ Pharm. Pharm. Sci..

[16] Allen T.M., Cullis P.R. (2013). Liposomal drug delivery systems: From concept to clinical applications. Adv. Drug Deliv. Rev..

[17] Bulbake U., Doppalapudi S., Kommineni N., Khan W. (2017). Liposomal formulations in clinical use: An updated review. Pharmaceutics.

[18] Torchilin V.P. (2005). Recent advances with liposomes as pharmaceutical carriers. Nat. Rev. Drug Discov..

[19] Heger Z., Polanska H., Rodrigo M.A.M., Guran R., Kulich P., Kopel P., Masarik M., Eckschlager T., Stiborova M., Kizek R. (2016). Prostate tumor attenuation in the nu/nu murine model due to anti-sarcosine antibodies in folate-targeted liposomes. Sci. Rep..

[20] Dothager R.S., Piwnica-Worms D. (2011). Nano in cancer: Linking chemistry, biology, and clinical applications in vivo. Cancer Res..

[21] Ali I., Salim K., Rather M.A., Wani W.A., Haque A. (2011). Advances in nano drugs for cancer chemotherapy. Curr. Cancer Drug Targ..

[22] Siegel R.L., Miller K.D., Jemal A. (2015). Cancer statistics, 2015. CA A Cancer J. Clin..

[23] Chen W.C., Completo G.C., Sigal D.S., Crocker P.R., Saven A., Paulson J.C. (2010). In vivo targeting of b-cell lymphoma with glycan ligands of cd22. Blood.

[24] Tirelli U., Errante D., Van Glabbeke M., Teodorovic I., Kluin-Nelemans J., Thomas J., Bron D., Rosti G., Somers R., Zagonel V. (1998). Chop is the standard regimen in patients > or= 70 years of age with intermediate-grade and high-grade non-hodgkin’s lymphoma: Results of a randomized study of the european organization for research and treatment of cancer lymphoma cooperative study group. J. Clin. Oncol..

[25] Mittal N.K., Bhattacharjee H., Mandal B., Balabathula P., Thoma L.A., Wood G.C. (2014). Targeted liposomal drug delivery systems for the treatment of b cell malignancies. J. Drug Targ..

[26] Binsky I., Haran M., Starlets D., Gore Y., Lantner F., Harpaz N., Leng L., Goldenberg D.M., Shvidel L., Berrebi A. (2007). Il-8 secreted in a macrophage migration-inhibitory factor- and cd74-dependent manner regulates b cell chronic lymphocytic leukemia survival. Proc. Natl. Acad. Sci. USA.

[27] DiJoseph J.F., Dougher M.M., Kalyandrug L.B., Armellino D.C., Boghaert E.R., Hamann P.R., Moran J.K., Damle N.K. (2006). Antitumor efficacy of a combination of cmc-544 (inotuzumab ozogamicin), a cd22-targeted cytotoxic immunoconjugate of calicheamicin, and rituximab against non-hodgkin’s b-cell lymphoma. Clin. Cancer Res..

[28] Du X., Beers R., Fitzgerald D.J., Pastan I. (2008). Differential cellular internalization of anti-cd19 and -cd22 immunotoxins results in different cytotoxic activity. Cancer Res.

[29] Loomis K., Smith B., Feng Y., Garg H., Yavlovich A., Campbell-Massa R., Dimitrov D.S., Blumenthal R., Xiao X., Puri A. (2010). Specific targeting to b cells by lipid-based nanoparticles conjugated with a novel cd22-scfv. Exp. Mol. Pathol..

[30] Sapra P., Allen T.M. (2004). Improved outcome when b-cell lymphoma is treated with combinations of immunoliposomal anticancer drugs targeted to both the cd19 and cd20 epitopes. Clin. Cancer Res..

[31] Tuscano J.M., Martin S.M., Ma Y., Zamboni W., O’Donnell R.T. (2010). Efficacy, biodistribution, and pharmacokinetics of cd22-targeted pegylated liposomal doxorubicin in a b-cell non–hodgkin’s lymphoma xenograft mouse model. Clin. Cancer Res..

[32] O’Donnell R.T., Martin S.M., Ma Y., Zamboni W.C., Tuscano J.M. (2010). Development and characterization of cd22-targeted pegylated-liposomal doxorubicin (il-pld). Investig. New Drugs.

[33] Sapra P., Allen T.M. (2002). Internalizing antibodies are necessary for improved therapeutic efficacy of antibody-targeted liposomal drugs. Cancer Res..

[34] Sapra P., Moase E.H., Ma J., Allen T.M. (2004). Improved therapeutic responses in a xenograft model of human b lymphoma (namalwa) for liposomal vincristine versus liposomal doxorubicin targeted via anti-cd19 igg2a or fab’ fragments. Clin. Cancer Res..

[35] Frishman W.H., Sung H.M., Yee H.C.M., Liu L.L., Einzig A.I., Dutcher J., Keefe D. (1996). Cardiovascular toxicity with cancer chemotherapy. Curr. Probl. Cardiol..

[36] Jensen B., Skovsgaard T., Nielsen S. (2002). Functional monitoring of anthracycline cardiotoxicity: A prospective, blinded, long-term observational study of outcome in 120 patients. Ann. Oncol..

[37] Speyer J., Wasserheit C. (1998). Strategies for reduction of anthracycline cardiac toxicity. Semin. Oncol..

[38] Binaschi M., Bigioni M., Cipollone A., Rossi C., Goso C., Maggi C.A., Capranico G., Animati F. (2001). Anthracyclines: Selected new developments. Curr. Med. Chem. Anti-Cancer Agents.

[39] Teicher B.A. (1996). Cancer Therapeutics: Experimental and Clinical Agents.

[40] He Y., Liu J., Durrant D., Yang H.S., Sweatman T., Lothstein L., Lee R.M. (2005). *N*-benzyladriamycin-14-valerate (AD198) induces apoptosis through protein kinase C-delta-induced phosphorylation of phospholipid scramblase 3. Cancer Res..

[41] Hofmann P.A., Israel M., Koseki Y., Laskin J., Gray J., Janik A., Sweatman T.W., Lothstein L. (2007). *N*-benzyladriamycin-14-valerate (AD 198): A non-cardiotoxic anthracycline that is cardioprotective through pkc-epsilon activation. J. Pharmacol. Exp. Ther..

[42] Cai C., Lothstein L., Morrison R.R., Hofmann P.A. (2010). Protection from doxorubicin-induced cardiomyopathy using the modified anthracycline *N*-benzyladriamycin-14-valerate (AD 198). J. Pharmacol. Exp. Ther..

[43] Lothstein L., Savranskaya L., Barrett C.M., Israel M., Sweatman T.W. (2006). *N*-benzyladriamycin-14-valerate (AD 198) activates protein kinase c-δ holoenzyme to trigger mitochondrial depolarization and cytochrome c release independently of permeability transition pore opening and Ca^2+^ influx. Anti-Cancer Drugs.

[44] Rathore K., Cekanova M. (2015). A novel derivative of doxorubicin, AD198, inhibits canine transitional cell carcinoma and osteosarcoma cells in vitro. Drug Des. Dev. Ther..

[45] Edwards S.K., Han Y., Liu Y., Kreider B.Z., Liu Y., Grewal S., Desai A., Baron J., Moore C.R., Luo C. (2016). Signaling mechanisms of bortezomib in traf3-deficient mouse b lymphoma and human multiple myeloma cells. Leuk. Res..

[46] Mittal N.K. (2015). Design, Development, Characterization and Testing of CD22 Targeted Long Circulating Liposomal Drug Delivery Systems for β Cell Malignancies. Ph.D. Thesis.

[47] Bangham A., Horne R. (1964). Negative staining of phospholipids and their structural modification by surface-active agents as observed in the electron microscope. J. Mol. Biol..

[48] Lopes de Menezes D.E., Pilarski L.M., Allen T.M. (1998). In vitro and in vivo targeting of immunoliposomal doxorubicin to human b-cell lymphoma. Cancer Res..

[49] Juliano R.L., Stamp D. (1975). The effect of particle size and charge on the clearance rates of liposomes and liposome encapsulated drugs. Biochem. Biophys. Res. Commun..

[50] Haran G., Cohen R., Bar L.K., Barenholz Y. (1993). Transmembrane ammonium sulfate gradients in liposomes produce efficient and stable entrapment of amphipathic weak bases. Biochim. Biophys. Acta-Biomembr..

[51] Lothstein L., Rodrigues P.J., Sweatman T.W., Israel M. (1998). Cytotoxicity and intracellular biotransformation of *N*-benzyladriamycin-14-yalerate (AD 198) are modulated by changes in 14-O-acyl chain length. Anti-Cancer Drugs.

[52] Stewart J.C.M. (1980). Colorimetric determination of phospholipids with ammonium ferrothiocyanate. Anal. Biochem..

[53] Zhang L., Chan J.M., Gu F.X., Rhee J.-W., Wang A.Z., Radovic-Moreno A.F., Alexis F., Langer R., Farokhzad O.C. (2008). Self-assembled lipid-polymer hybrid nanoparticles: A robust drug delivery platform. ACS Nano.

[54] Chan J.M., Zhang L., Yuet K.P., Liao G., Rhee J.-W., Langer R., Farokhzad O.C. (2009). PLGA-lecithin-PEG core-shell nanoparticles for controlled drug delivery. Biomaterials.

[55] Zhang L., Radovic-Moreno A.F., Alexis F., Gu F.X., Basto P.A., Bagalkot V., Jon S., Langer R.S., Farokhzad O.C. (2007). Co-delivery of hydrophobic and hydrophilic drugs from nanoparticle–aptamer bioconjugates. ChemMedChem.

[56] Oliveira S., Schiffelers R.M., van der Veeken J., van der Meel R., Vongpromek R., en Henegouwen P.M.V.B., Storm G., Roovers R.C. (2010). Downregulation of EGFR by a novel multivalent nanobody-liposome platform. J. Control. Release.

[57] Huth U.S., Schubert R., Peschka-Süss R. (2006). Investigating the uptake and intracellular fate of pH-sensitive liposomes by flow cytometry and spectral bio-imaging. J. Control. Release.

[58] Mosmann T. (1983). Rapid colorimetric assay for cellular growth and survival: Application to proliferation and cytotoxicity assays. J. Immunol. Methods.

[59] Douglas K.L., Piccirillo C.A., Tabrizian M. (2008). Cell line-dependent internalization pathways and intracellular trafficking determine transfection efficiency of nanoparticle vectors. Eur. J. Pharm. Biopharm..

[60] Gao H., Yang Z., Zhang S., Cao S., Shen S., Pang Z., Jiang X. (2013). Ligand modified nanoparticles increases cell uptake, alters endocytosis and elevates glioma distribution and internalization. Sci. Rep..

[61] Suresh D., Zambre A., Chanda N., Hoffman T.J., Smith C.J., Robertson J.D., Kannan R. (2014). Bombesin peptide conjugated gold nanocages internalize via clathrin mediated endocytosis. Bioconjug. Chem..

[62] Päuser S., Reszka R., Wagner S., Wolf K.J., Buhr H.J., Berger G. (1997). Liposome-encapsulated superparamagnetic iron oxide particles as markers in an MRI-guided search for tumor-specific drug carriers. Anticancer Drug Des..

[63] Wu L., Yu X., Feizpour A., Reinhard B.M. (2014). Nanoconjugation: A materials approach to enhance epidermal growth factor induced apoptosis. Biomater. Sci..

[64] Jiang M., Gan L., Zhu C., Dong Y., Liu J., Gan Y. (2012). Cationic core-shell liponanoparticles for ocular gene delivery. Biomaterials.

[65] Zhao Z., Lou S., Hu Y., Zhu J., Zhang C. (2017). A nano-in-nano polymer–dendrimer nanoparticle-based nanosystem for controlled multidrug delivery. Mol. Pharm..

[66] Jaggi M., Rao P.S., Smith D.J., Wheelock M.J., Johnson K.R., Hemstreet G.P., Balaji K. (2005). E-cadherin phosphorylation by protein kinase D1/protein kinase Cμ is associated with altered cellular aggregation and motility in prostate cancer. Cancer Res..

[67] Lian T., Ho R.J. (2001). Trends and developments in liposome drug delivery systems. J. Pharm. Sci..

[68] Gregoriadis G., Senior J. (1980). The phospholipid component of small unilamellar liposomes controls the rate of clearance of entrapped solutes from the circulation. FEBS Lett..

[69] Senior J.H. (1987). Fate and behavior of liposomes in vivo: A review of controlling factors. Crit. Rev. Ther. Drug Carr. Syst..

[70] Drummond D.C., Meyer O., Hong K., Kirpotin D.B., Papahadjopoulos D. (1999). Optimizing liposomes for delivery of chemotherapeutic agents to solid tumors. Pharmacol. Rev..

[71] Hu Y., Hoerle R., Ehrich M., Zhang C. (2015). Engineering the lipid layer of lipid–PLGA hybrid nanoparticles for enhanced in vitro cellular uptake and improved stability. Acta Biomater..

[72] Woodle M.C., Matthay K.K., Newman M.S., Hidayat J.E., Collins L.R., Redemann C., Martin F.J., Papahadjopoulos D. (1992). Versatility in lipid compositions showing prolonged circulation with sterically stabilized liposomes. Biochim. Biophys. Acta.

[73] Gramse G., Dols-Perez A., Edwards M.A., Fumagalli L., Gomila G. (2013). Nanoscale measurement of the dielectric constant of supported lipid bilayers in aqueous solutions with electrostatic force microscopy. Biophys. J..

[74] Torre L.G., Carneiro A.L., Rosada R.S., Silva C.L., Santana M.H.A. (2007). A mathematical model describing the kinetic of cationic liposome production from dried lipid films adsorbed in a multitubular system. Br. J. Chem. Eng..

[75] Budha N.R., Lee R.B., Hurdle J.G., Lee R.E., Meibohm B. (2009). A simple in vitro PK/PD model system to determine time–kill curves of drugs against mycobacteria. Tuberculosis.

[76] Schmid S.L., Carter L.L. (1990). ATP is required for receptor-mediated endocytosis in intact cells. J. Cell Biol..

[77] Kou L., Sun J., Zhai Y., He Z. (2013). The endocytosis and intracellular fate of nanomedicines: Implication for rational design. Asian J. Pharm. Sci..

[78] Luzio J.P., Pryor P.R., Bright N.A. (2007). Lysosomes: Fusion and function. Nat. Rev. Mol. Cell Biol..

[79] Finver S.N., Nishikura K., Finger L.R., Haluska F.G., Finan J., Nowell P.C., Croce C.M. (1988). Sequence analysis of the MYC oncogene involved in the t(8;14)(q24;q11) chromosome translocation in a human leukemia T-cell line indicates that putative regulatory regions are not altered. Proc. Natl. Acad. Sci. USA.

[80] Nicholson K.M., Anderson N.G. (2002). The protein kinase B/Akt signalling pathway in human malignancy. Cell. Signal..

[81] Sarbassov D.D., Guertin D.A., Ali S.M., Sabatini D.M. (2005). Phosphorylation and regulation of Akt/PKB by the rictor-mtor complex. Science.

[82] Edwards S.K., Moore C.R., Liu Y., Grewal S., Covey L.R., Xie P. (2013). *N*-benzyladriamycin-14-valerate (AD 198) exhibits potent anti-tumor activity on TRAF3-deficient mouse b lymphoma and human multiple myeloma. BMC Cancer.

[83] Bubici C., Papa S. (2014). Jnk signalling in cancer: In need of new, smarter therapeutic targets. Br. J. Pharmacol..

